# Emerging horizons: clinical applications and multifaceted benefits of SGLT-2 inhibitors beyond diabetes

**DOI:** 10.3389/fcvm.2025.1482918

**Published:** 2025-03-20

**Authors:** Qing Feng, Miaoqiong Wu, Zizhao Mai

**Affiliations:** ^1^ Department of Cardiology, Kaiping Central Hospital, Kaiping, China; ^2^ Department of Endocrinology, Kaiping Central Hospital, Kaiping, China; ^3^ School of Stomatology, Stomatological Hospital, Southern Medical University, Guangzhou, Guangdong, China

**Keywords:** SGLT-2 inhibitors, cardiovascular diseases, heart failure, chronic kidney disease, nonalcoholic fatty liver disease

## Abstract

SGLT-2 inhibitors, initially developed for type 2 diabetes, demonstrate profound cardiorenal and metabolic benefits. This review synthesizes evidence from clinical trials and mechanistic studies to elucidate their roles in cardiovascular diseases, chronic kidney disease, and non-alcoholic fatty liver disease. Key findings include a notable reduction in cardiovascular death/heart failure hospitalization, a marked decrease in heart failure hospitalization risk, and significant improvements in renal and hepatic outcomes. Emerging mechanisms, such as autophagy induction, ketone utilization, and anti-inflammatory effects, underpin these benefits. Ongoing trials explore their potential in non-diabetic populations, positioning SGLT-2 inhibitors as transformative agents in multisystem disease management.

## 1 Introduction

Sodium-glucose cotransporter 2 inhibitors (SGLT-2i), initially developed for managing type 2 diabetes mellitus (T2DM), have garnered significant attention for their multifaceted therapeutic benefits extending beyond glycemic control (1). These inhibitors, including canagliflozin, dapagliflozin, and empagliflozin, function by inhibiting the SGLT-2 protein in the proximal tubules of the kidney, thereby promoting glucose excretion through urine. This mechanism not only lowers blood glucose levels but also imparts several cardiovascular, renal, and metabolic benefits (2).

The journey of SGLT-2i dates back to the late 19th century with the discovery of phlorizin, a natural compound with glucose-lowering properties (3). Despite its initial promise, phlorizin's non-selective nature and gastrointestinal side effects hindered its clinical application (4). Advances in medicinal chemistry in the late 20th and early 21st centuries led to the development of selective SGLT-2i, revolutionizing the treatment landscape for T2DM and paving the way for their expanded use in cardiovascular and renal disease management (5, 6). Beyond T2DM, the current clinical indications for SGLT-2i mainly include heart failure (HF) and chronic kidney disease (CKD).

This review innovatively summarizes the potential clinical applications and mechanisms of SGLT-2i beyond T2DM, including HF, and CKD, myocardial infarction (MI), hypertension, arrhythmias, and non-alcoholic fatty liver disease (NAFLD). By examining the clinical evidence and exploring these mechanisms, we seek to highlight the transformative potential of SGLT-2i in managing these diseases and improving patient outcomes.

## 2 SGLT-2 physiology and origins of SGLT-2i

SGLT-2 protein is expressed in the proximal tubules of the kidney, where it is responsible for reabsorbing approximately 90% of filtered glucose. In patients with T2DM, SGLT-2 expression may be upregulated, exacerbating hyperglycemia (7). SGLT-2i are a class of medications initially developed for the treatment of T2DM (8). These drugs function by inhibiting the SGLT-2 protein in the proximal tubules of the kidney, thereby reducing the reabsorption of glucose and sodium (9). This action promotes the excretion of glucose through urine, leading to lower blood glucose levels (10).

The origins of SGLT-2i date back to the 1980s, with the study of the natural compound phlorizin, which inhibits both SGLT1 and SGLT2. However, due to its non-selective inhibition, phlorizin exhibited gastrointestinal instability and undesirable side effects, limiting its therapeutic potential. In the 1990s, as the understanding of glucose transport in the kidneys deepened, SGLT2 was identified as the key protein responsible for reabsorbing most of the glucose in the renal proximal tubules. This discovery laid the foundation for the development of selective SGLT-2i (11).

In the early 2000s, pharmaceutical chemists began creating selective SGLT-2i with the goal of reducing side effects and enhancing efficacy. This effort led to the development of drugs such as dapagliflozin, empagliflozin, and canagliflozin. Dapagliflozin became the first SGLT-2i to receive approval, launching in Europe in 2012 and subsequently being approved by the Food and Drug Administration in 2013 for the treatment of T2DM (12). Empagliflozin and canagliflozin followed, and clinical trials demonstrated their effectiveness not only in lowering blood glucose but also in promoting weight loss and reducing blood pressure (BP) (8).

**Figure 1:**
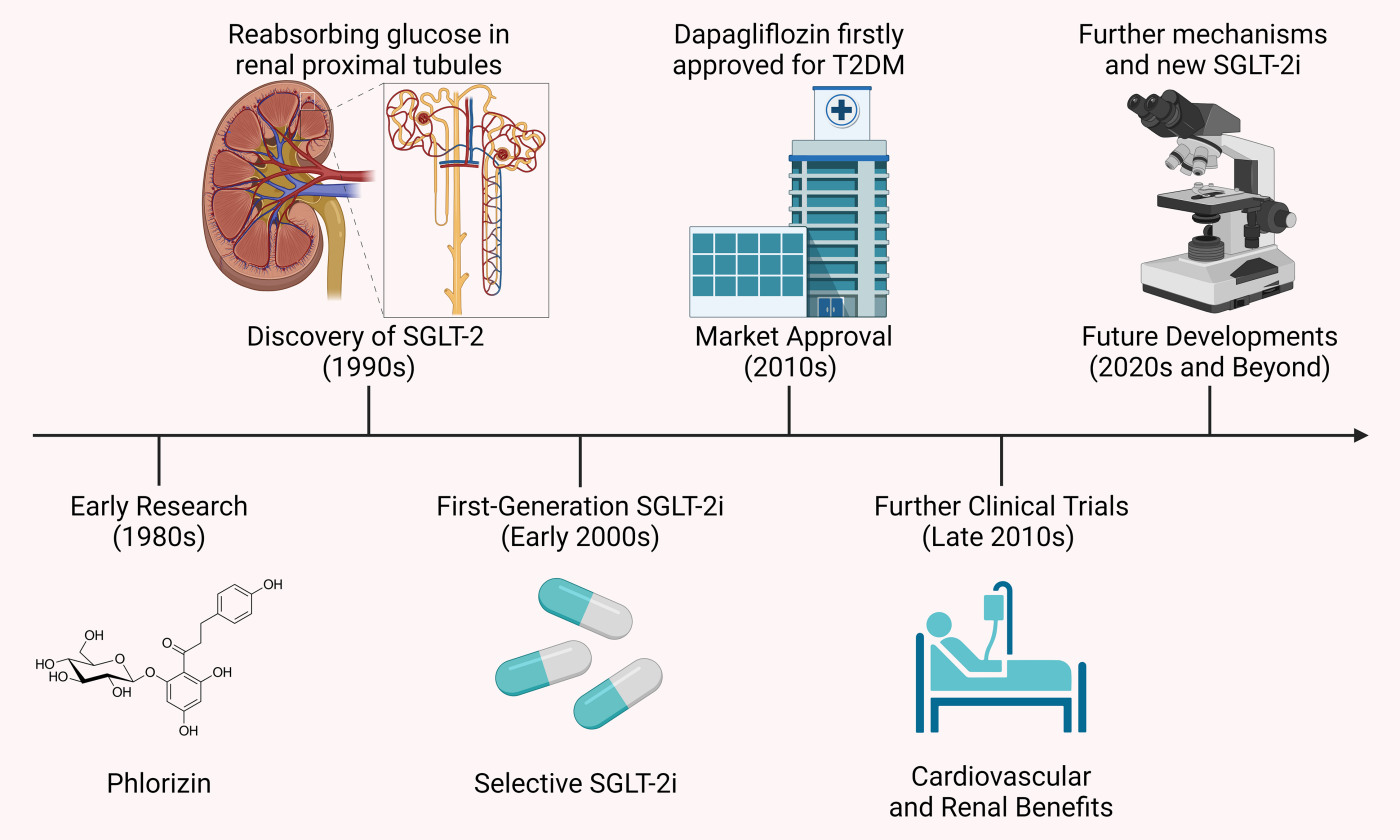
Timeline of SGLT-2i development and key milestones. The development of SGLT-2i began in the 1980s with phlorizin, a compound that inhibited both SGLT-1 and SGLT-2 but had unsuitable gastrointestinal side effects. The 1990s discovery of the SGLT-2 protein, key to glucose reabsorption in the kidney, led to selective inhibitors like dapagliflozin and canagliflozin, approved in the early 2010s. Clinical studies later showed these drugs not only lower blood glucose but also offer cardiovascular and renal protection, extending their use to HF and CKD.

Further clinical studies in the late 2010s revealed that SGLT-2i offered significant cardiovascular and renal protective effects, particularly in diabetic patients with existing cardiovascular disease or at high risk. This led to the expansion of their indications to include HF and CKD (13). With a growing understanding of their mechanisms, SGLT-2i are now being explored for use in non-diabetic patients, especially in the areas of cardiovascular and renal diseases. Additionally, new SGLT-2i are being developed to further improve efficacy and reduce side effects. Figure 1 presents a timeline of the development of SGLT-2i and key milestones.

## 3 SGLT-2i: revolutionizing cardiovascular diseases (CVDs) management

SGLT-2i have significantly impacted the landscape of CVDs management, extending their benefits far beyond glycemic control in diabetes. These inhibitors, including empagliflozin, dapagliflozin, canagliflozin, and sotagliflozin, have demonstrated profound cardioprotective effects, notably in HF management. Clinical trials have consistently shown that SGLT-2i reduce HF-related morbidity and mortality, improve cardiac outcomes, and enhance the quality of life for patients with HF (14). Moreover, these drugs also offer benefits in the context of MI, hypertension, and arrhythmias, highlighting their multifaceted role in CVDs treatment.

**Figure 2:**
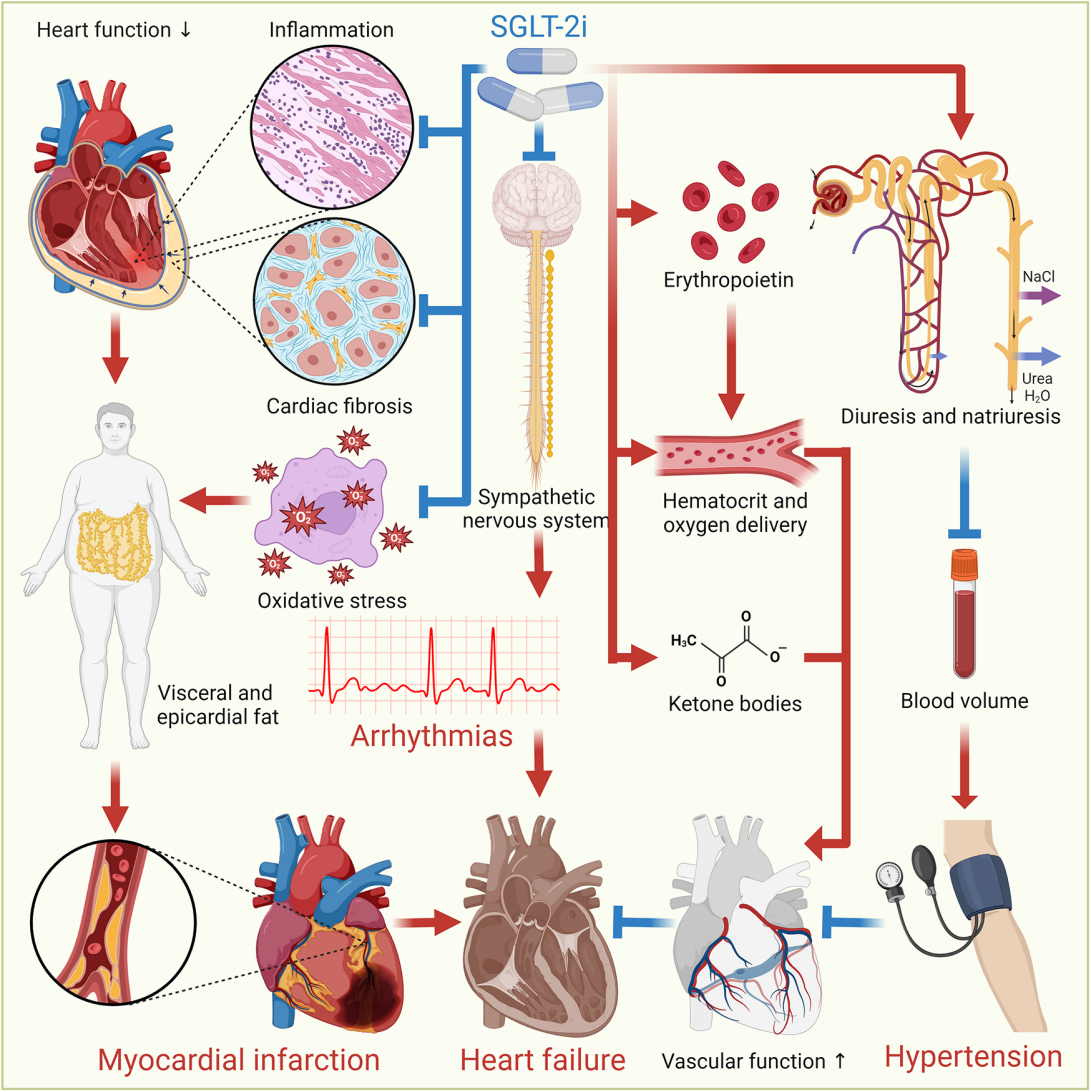
Cardiovascular benefits of SGLT-2i. SGLT-2i offer cardiovascular benefits through various mechanisms. They induce autophagy, reduce oxidative stress and inflammation, and decrease visceral and epicardial fat. They also promote osmotic diuresis and natriuresis, reducing BP and sodium levels, aiding HF management. Additionally, SGLT-2i enhance erythropoietin production, improving hematocrit and oxygen delivery, and increase ketone bodies for better cardiac energetics. They inhibit the sympathetic nervous system, modulate cardiac inflammation and fibrosis, promote reverse cardiac remodeling, and improve vascular function, collectively enhancing heart health.

SGLT-2i offer cardiovascular benefits through a range of mechanisms, despite SGLT-2 not being significantly expressed in cardiomyocytes. They indirectly modulate cardiac function via systemic effects, such as improved myocardial energetics through ketone utilization, reduced oxidative stress, and inhibition of the Na^+^/H^+^ exchanger 1, which mitigates intracellular Na^+^ overload, calcium dysregulation, and calcineurin-driven cell death (15). SGLT-2i induce glycosuria, lowering the portal insulin-to-glucagon ratio and mimicking prolonged fasting, which stimulates lipolysis, hepatic ketogenesis, and increased circulating ketones (16). This shift in myocardial metabolism from glucose to more energy-efficient substrates, like ketone bodies, fatty acids, and branched-chain amino acids, is supported by altered enzyme activity favoring fatty acids/ketone/branched-chain amino acids oxidation while reducing glucose utilization (17). Additionally, SGLT-2i attenuate cardiac fibrosis by suppressing oxidative stress, collagen synthesis, and fibroblast activation (18). They also activate AMPK to enhance bioenergetics, promote macrophage polarization toward anti-inflammatory M2 phenotypes, and reduce CaMKII-mediated sarcoplasmic reticulum Ca^2+^ leaks (19). These effects are independent of glycemic control, as cardiac SGLT2 receptors are absent, highlighting the pleiotropic benefits of SGLT-2i through metabolic reprogramming, anti-fibrotic actions, and improved cardiomyocyte Ca^2+^/Na^+^ homeostasis. Furthermore, SGLT-2i promote a fasting-like response by stimulating autophagy, reducing visceral and epicardial fat, lowering BP, and aiding in HF management through osmotic diuresis and natriuresis (20). They also stimulate erythropoietin production to enhance hematocrit and oxygen delivery, improve cardiac energetics by increasing ketone bodies, inhibit the sympathetic nervous system, modulate cardiac inflammation and fibrosis, and promote reverse cardiac remodeling, all contributing to improved heart health (2). Figure 2 illustrates the mechanisms through which SGLT-2i provide cardiovascular benefits.

Given these wide-ranging benefits, SGLT-2i have become a cornerstone in the therapeutic arsenal for managing various cardiovascular conditions. Future research is poised to explore additional indications and long-term benefits of these inhibitors, potentially broadening their application in CVD management.

### 3.1 Clinical applications and mechanisms of SGLT-2i for HF

HF is the terminal manifestation of almost all CVDs. HF is a complex clinical syndrome characterized by any structural or functional impairment of ventricular filling or blood ejection (21). Ischemic heart disease, the leading cause of death worldwide, is also the primary cause of HF (22). HF is a common global disease with high morbidity and mortality rates (23). It is estimated that 26 million people worldwide suffer from HF, which increases healthcare costs, reduces functional capacity, and severely impacts quality of life (24). HF is characterized by impaired myocardial function leading to inadequate blood supply to the body. Patients typically present with fatigue, dyspnea, reduced exercise tolerance, and congestion in the systemic or pulmonary circulation. General treatment aims to relieve systemic and pulmonary congestion and stabilize hemodynamic status (25).

The cardioprotective effects of SGLT-2i in HF are multifaceted. These drugs promote glucosuria, leading to reduced blood glucose levels and consequently lower insulin resistance and weight reduction, which alleviates the cardiac workload (26). SGLT-2i also enhance natriuresis and diuresis, which help to reduce BP and decrease the volume overload on the heart (27). Additionally, these inhibitors improve myocardial energy metabolism by increasing ketone body utilization and reducing myocardial inflammation and oxidative stress, thereby preserving cardiac function and structure (28). The modulation of neurohormonal pathways, including the reduction of sympathetic nervous system activity and improvement in renal function, further contributes to their therapeutic benefits in HF (29).

SGLT-2i have demonstrated significant benefits in the management of HF, addressing various patient populations with differing HF characteristics. The effects and mechanisms of SGLT-2i vary depending on the ejection fraction (EF) of HF patients, leading to differential therapeutic outcomes across HF subtypes. In HF with reduced EF (HFrEF), dapagliflozin improved Kansas City Cardiomyopathy Questionnaire (KCCQ) scores and significantly reduced the risk of worsening HF or cardiovascular death, while empagliflozin also lowered the risk of cardiovascular death or HF hospitalization but did not improve cardiac energetics or circulating serum metabolites related to energy metabolism (30–32). In HFrEF patients without diabetes, empagliflozin improved adiposity, interstitial myocardial fibrosis, aortic stiffness, and inflammatory markers (33). For HF with preserved EF (HFpEF), dapagliflozin did not enhance KCCQ scores or six-minute walking distance but significantly improved patient-reported symptoms, physical limitations, and exercise function, while also reducing resting and exercise pulmonary capillary wedge pressure, plasma volume, and body weight. In HFpEF or HF with mid-range EF (HFmrEF), both dapagliflozin and empagliflozin reduced the combined risk of worsening HF or cardiovascular death (34, 35).

The therapeutic effects of SGLT-2i also vary depending on the progression of HF. In patients with acute HF, dapagliflozin initiation has been shown to be safe and fulfills a component of guideline-directed medical therapy optimization (36). Empagliflozin, during acute decompensated HF, resulted in higher circulating ketone body concentrations, particularly acetone, compared to stabilized phases (37). It also produced clinical benefits and was well tolerated regardless of background mineralocorticoid receptor antagonist use (38). For patients with chronic HF and T2DM, empagliflozin increased 24 h urine volume without an increase in urinary sodium when combined with loop diuretics, highlighting its renal and cardiovascular effects (39). More broadly, SGLT-2i significantly reduced the risk of cardiovascular death or hospitalization for HF by 23%, and hospitalization for HF by 31% (40). Table 1 reviews clinical trials evaluating the effects of SGLT-2i in HF.

**Table 1:** Overview of clinical trials investigating SGLT-2i in HF.

Disease characteristic	Primary finding	Measurement parameters	SGLT-2i Dose	Follow-up duration	Patients enrolled	Clinical trial number	Refs.
HF	Canagliflozin significantly improves symptom burden in HF, regardless of EF or diabetes status.	KCQQ scores	Canagliflozin 100 mg/d	12 weeks	476	NCT04252287	(14)
HF	Empagliflozin significantly reduced pulmonary artery pressures independent of loop diuretic management.	PA diastolic pressure, PA systolic pressure, PA mean pressure, KCQQ scores, natriuretic peptide levels, 6-minute walk distance (6MWD)	Empagliflozin 10 mg/d	12 weeks	65	NCT03030222	(117)
HFrEF or HFpEF	Dapagliflozin improved KCCQ scores in HFrEF patients but did not improve KCCQ scores or 6MWD; no improvement in HFpEF patients.	KCCQ scores, 6MWD	Dapagliflozin 10 mg/d	16 weeks	817	NCT03877237, NCT03877224	(30)
HFrEF or HFpEF	Empagliflozin did not improve cardiac energetics or change circulating serum metabolites associated with energy metabolism.	Cardiac phosphocreatine ratio, serum metabolomics, circulating ketone bodies	Empagliflozin 10 mg/d	12 weeks	72	NCT03332212	(118)
HFrEF without diabetes	Empagliflozin significantly improved adiposity, interstitial myocardial fibrosis, aortic stiffness, and inflammatory markers.	Epicardial adipose tissue volume, interstitial myocardial fibrosis, aortic stiffness, subcutaneous adipose tissue area, extracellular volume, matrix volume, cardiomyocyte volume, pulsed wave velocity, inflammatory biomarkers	Empagliflozin 10 mg/d	6 months	80	NCT03485222	(33)
HFrEF	Empagliflozin significantly reduced the risk of cardiovascular death or hospitalization for HF.	Cardiovascular death, hospitalization for HF, eGFR	Empagliflozin 10 mg/d	16 months (median)	3,730	NCT03057977	(31)
HFrEF	Dapagliflozin reduced the risk of worsening HF or death from cardiovascular causes.	Worsening HF, cardiovascular death, all-cause mortality, AEs	Dapagliflozin 10 mg/d	18.2 months (median)	4,744	NCT03036124	(32)
HFpEF	Dapagliflozin significantly improved patient-reported symptoms, physical limitations, and exercise function.	KCQQ scores, six-minute walking distance, weight, natriuretic peptides, glycated hemoglobin, SBP	Dapagliflozin 10 mg/d	12 weeks	324	NCT03030235	(119)
HFpEF	Dapagliflozin reduced resting and exercise pulmonary capillary wedge pressure, along with favorable effects on plasma volume and body weight.	Pulmonary capillary wedge pressure, body weight, plasma volume, oxygen consumption, arterial lactate	Dapagliflozin 10 mg/d	24 weeks	38	NCT04730947	(120)
HFpEF or HFmrEF	Empagliflozin reduced the combined risk of cardiovascular death or hospitalization for HF, regardless of diabetes.	Cardiovascular death, hospitalization for HF, total hospitalizations for HF	Empagliflozin 10 mg/d	26.2 months (median)	5,988	NCT03057951	(34)
HFpEF or HFmrEF	Dapagliflozin reduced the combined risk of worsening HF or cardiovascular death.	Worsening HF, cardiovascular death, total events, symptom burden	Dapagliflozin 10 mg/d	2.3 years (median)	6,263	NCT03619213	(35)
Acute HF	Early dapagliflozin initiation is safe and fulfills a component of Guideline-Directed Medical Therapy optimization.	Diuretic efficiency, 24 h natriuresis, urine output	Dapagliflozin 10 mg/d	5 days or hospital discharge	240	NCT04298229	(36)
Acute HF	Circulating ketone body concentrations, especially acetone, were higher during acute decompensated HF compared with after stabilization.	Plasma concentrations of ketone bodies, NT-proBNP	Empagliflozin 10 mg/d	30 days	79	NCT03200860	(37)
Acute HF	Empagliflozin produced clinical benefit and was well tolerated irrespective of background mineralocorticoid receptor antagonist use.	All-cause death, HF events, KCQQ scores, first HF hospitalization, cardiovascular death	Empagliflozin 10 mg/d	90 days	530	NCT04157751	(38)
Chronic HF	Empagliflozin caused a significant increase in 24 h urine volume without an increase in urinary sodium when used in combination with loop diuretic.	24 h urinary volume, 24 h natriuresis, fractional excretion of sodium, electrolyte-free water clearance, body weight, serum urate	Empagliflozin 25 mg/d	6 weeks	23	NCT03226457	(39)

In summary, SGLT-2i have shown diverse and significant clinical benefits in various HF scenarios. These benefits span improvements in symptoms, reduction in cardiovascular events, and optimization of HF management strategies, making them a crucial component in contemporary HF treatment regimens.

### 3.2 Potential clinical applications and mechanisms of SGLT-2i for MI

MI, commonly known as a “heart attack”, is caused by the partial or complete cessation of blood flow to a part of the heart muscle (41). MI can range from being asymptomatic to causing catastrophic events like hemodynamic deterioration and sudden death. Most MIs are triggered by underlying coronary artery disease, where the occlusion of a coronary artery leads to myocardial hypoxia. Prolonged oxygen deprivation results in the death and necrosis of myocardial cells. Patients may experience chest discomfort or pressure that can radiate to the neck, jaw, shoulders, or arms (42). In addition to the patient's medical history and physical examination, myocardial ischemia is evidenced by changes on an electrocardiogram and elevated biochemical markers such as cardiac troponins (43).

SGLT-2i offer cardioprotective effects against MI through multiple mechanisms. These include the suppression of autophagic cell death by inhibiting the Na^+^/H^+^ exchanger 1, reducing infarct size and myocardial fibrosis (44). Furthermore, these inhibitors help normalize mitochondrial size and number, and prevent excessive reduction in mitochondrial size after MI, leading to improved structural and functional heart outcomes post-MI (45). In addition, they have been shown to attenuate cardiac fibrosis by regulating macrophage polarization through a STAT3-dependent pathway, which helps in reducing adverse ventricular remodeling post-MI (46). They also exhibit significant anti-inflammatory effects, lowering levels of inflammatory markers and contributing to smaller infarct sizes (47). Additionally, SGLT-2i reduce the risk of new-onset cardiac arrhythmias, such as atrial fibrillation (AF) and ventricular arrhythmias, during hospitalization for acute MI (48).

SGLT-2i have shown considerable promise in the context of MI, addressing various patient populations and clinical scenarios. In patients with acute MI, the EMMY trial demonstrated that empagliflozin was associated with a significantly greater N-terminal pro-hormone of brain natriuretic peptide (NT-proBNP) reduction over 26 weeks, alongside notable improvements in echocardiographic functional and structural parameters (49). For patients without diabetes or established HF presenting with MI and impaired left ventricular (LV) systolic function or Q-wave MI, the DAPA-MI trial explores the potential of dapagliflozin to further improve outcomes in these patients (50). Similarly, dapagliflozin showed significant benefits in cardiometabolic outcomes in patients with acute MI without prior diabetes or chronic HF. In patients with T2DM and a history of MI, dapagliflozin robustly reduced the risk of major adverse cardiovascular events (MACE) and cardiovascular death or hospitalization for HF (51). Additionally, in patients with acute MI and newly reduced LVEF below 45% or congestion, empagliflozin reduced first and total HF hospitalizations across the range of LVEF with or without congestion (52).

Empagliflozin also showed promise in reducing the risk of first and recurrent HF events in patients with acute MI at risk for HF due to newly developed LVEF below 45% or signs of congestion, as seen in the EMPACT-MI trial (53). Early administration of SGLT-2i in acute MI patients with T2DM, as investigated in the EMBODY trial, might effectively improve cardiac nerve activity without adverse events (AEs) (54). For non-diabetic patients with anterior ST-elevation MI and LVEF below 50%, dapagliflozin appears to play a role in preventing LV dysfunction and maintaining cardiac function, as demonstrated in the DACAMI trial (55). Furthermore, in patients with T2DM and CVD, empagliflozin reduced the risk of total MI events by 21%, driven by effects on both type 1 and type 2 MIs, according to insights from the EMPA-REG OUTCOME trial (56). Table 2 presents clinical trials investigating the use of SGLT-2i in MI.

**Table 2:** Overview of clinical trials investigating SGLT-2i in MI.

Disease characteristic	Primary finding	Measurement parameters	SGLT-2i dose	Follow-up duration	Patients enrolled	Clinical trial number	Refs.
Acute MI without prior diabetes or chronic HF	Significant benefits with regard to improvement in cardiometabolic outcomes but no impact on the composite of cardiovascular death or hospitalization for HF compared with placebo.	Death, hospitalization for HF, nonfatal MI, AF/AFL, T2DM, NYHA Functional Classification, body weight decrease ≥5%	Dapagliflozin 10 mg/d	Approximately 1 year	4,017	NCT04564742	(121)
MI and impaired LV systolic function or Q-wave MI without diabetes or established HF	The trial explores opportunities to improve further the outcome of patients with impaired LV function after MI using dapagliflozin.	Death, hospitalization for HF, nonfatal MI, AF/AFL, new onset of T2DM, HF symptoms, body weight decrease ≥5%	Dapagliflozin 10 mg/d	2.5 years	4,000	NCT04564742	(50)
Anterior ST-elevation MI and LVEF <50% without diabetic	Dapagliflozin appears to have a role in preventing LV dysfunction and maintaining cardiac function following anterior ST-elevation MI.	NT-proBNP, LVEF, LV diastolic dimension, LV mass index	Dapagliflozin 10 mg/d	12 weeks	100	NCT05424315	(55)
Acute MI and newly reduced LVEF <45%, congestion, or both	Empagliflozin reduced first and total HF hospitalizations across the range of LVEF with and without congestion.	LVEF, congestion, first and total HF hospitalizations, all-cause death	Empagliflozin 10 mg/d	17.9 months (median)	6,522	NCT04509674	(52, 53)
Acute MI	Empagliflozin was associated with a significantly greater NT-proBNP reduction over 26 weeks, accompanied by a significant improvement in echocardiographic functional and structural parameters.	NT-proBNP, echocardiographic parameters, LVEF, E/e’ ratio, LV end-systolic and end-diastolic volumes	Empagliflozin 10 mg/d	26 weeks	476	NCT03087773	(49)
Acute MI	Treatment with empagliflozin did not lead to a significantly lower risk of a first hospitalization for HF or death from any cause than placebo.	Hospitalization for HF, death from any cause	Empagliflozin 10 mg/d	17.9 months (median)	6,522	NCT04509674	(122)
Acute MI	Early SGLT-2i administration in acute MI patients might be effective in improving cardiac nerve activity without any AEs.	Heart rate variability, heart rate turbulence, standard deviation of all 5-min mean normal RR intervals, low-frequency-to-high-frequency ratio	Empagliflozin 10 mg/d	24 weeks	105	UMIN000030158	(54)
Atherosclerotic CVD	Dapagliflozin robustly reduced the risk of MACE and cardiovascular death/hospitalization for HF in patients with previous MI.	MACE, cardiovascular death, hospitalization for HF	Dapagliflozin 10 mg/d	4.2 years	17,160	NCT01730534	(51)
Atherosclerotic CVD	Empagliflozin reduced the risk of total MI events by 21%, driven by effects on type 1 and type 2 MIs.	Total MI events, fatal and non-fatal MI, types of MI	Empagliflozin 10 mg/d	3.1 years (median)	7,020	NCT01131676	(56)

In brief, SGLT-2i like empagliflozin and dapagliflozin have shown diverse benefits in managing MI, particularly in improving cardiac function, reducing cardiovascular events, and optimizing HF outcomes in various patient populations.

### 3.3 Potential clinical applications and mechanisms of SGLT-2i for hypertension

Hypertension, defined as BP consistently at or above 140/90 mmHg, affects an estimated 1.28 billion adults worldwide, with the two-thirds residing in low- and middle-income countries (57). It is one of the most significant risk factors for ischemic heart disease, stroke, other CVDs, CKD, and dementia. Key risk factors for hypertension include advanced age, genetics, obesity, physical inactivity, high-salt diet, and excessive alcohol consumption (58). While most individuals with hypertension exhibit no symptoms, severely elevated BP can cause headaches, blurred vision, and chest pain. Hypertension remains the leading preventable cause of CVD-related deaths and disease burden globally and in most regions of the world (59).

The antihypertensive effects of SGLT-2i are mediated through several mechanisms. These drugs induce osmotic diuresis and natriuresis, leading to reduced blood volume and decreased preload on the heart (60). By inhibiting sodium reabsorption in the proximal tubules, SGLT-2i promote sodium excretion, which helps lower BP (61). Additionally, these inhibitors improve endothelial function and reduce arterial stiffness, which are critical factors in the pathogenesis of hypertension (62). Furthermore, they inhibit the renin-angiotensin-aldosterone system (RAAS) and decrease oxidative stress and inflammation in renal tissues, mitigating hypertension and protecting against renal and cardiovascular damage (61, 63).

SGLT-2i have demonstrated considerable efficacy in managing hypertension, especially in patients with T2DM. Clinical trials underscore their significant impact on BP and broader cardiovascular outcomes. For instance, empagliflozin has yielded substantial and clinically meaningful reductions in both BP and HbA1c levels among T2DM patients with hypertension, while maintaining a good tolerance profile (64). This highlights its dual benefit in simultaneously controlling glucose levels and BP. Over periods ranging from 12 to 24 weeks, empagliflozin consistently decreased glycohemoglobin, body weight, and BP, marking its prolonged efficacy in this patient group (65). Additionally, it has significantly lowered mean 24 h systolic BP (SBP) in both dippers and non-dippers, demonstrating versatility across different BP profiles (66). Empagliflozin also benefits T2DM patients on various antihypertensive treatments, including those on multiple drugs, diuretics, or ACE inhibitors/ARBs, effectively reducing both SBP and diastolic BP (DBP) (67). Notably, in elderly patients with T2DM and hypertension, it has notably decreased BP, improved endothelial function, and reduced arterial stiffness, suggesting additional cardiovascular protective effects (68). For those receiving renin-angiotensin blockers, dapagliflozin has significantly reduced albuminuria, a critical marker of kidney damage, with these benefits persisting even after adjusting for changes in HbA1c, BP, body weight, and estimated glomerular filtration rate (eGFR), underlining its renal protective properties in hypertensive diabetic patients (69). Table 3 covers clinical trials examining the role of SGLT-2i in hypertension.

**Table 3:** Overview of potential clinical trials investigating SGLT-2i in hypertension.

Disease characteristic	Primary finding	Measurement parameters	SGLT-2i dose	Follow-up duration	Patients enrolled	Clinical trial number	Refs.
Hypertension	Empagliflozin was associated with significant and clinically meaningful reductions in BP and HbA1c versus placebo and was well tolerated.	Mean 24 h SBP, mean 24 h DBP, office BP, HbA1c	Empagliflozin 10 or 25 mg/d	12 weeks	825	NCT01370005	(64)
Hypertension	Empagliflozin significantly reduced glycohemoglobin, body weight, and BP, with BP effects increasing from 12 to 24 weeks.	Glycohemoglobin, office BP, 24 h ambulatory BP, body weight	Empagliflozin 10 mg (first 4 weeks), then 25 mg/d	24 weeks	150	NCT02182830	(65)
Hypertension	Empagliflozin significantly reduced mean 24 h SBP compared with placebo in both dippers and non-dippers.	Mean 24 h SBP, mean 24 h DBP, heart rate	Empagliflozin 10 or 25 mg/d	12 weeks	767	Not specified	(66)
Hypertension	Empagliflozin significantly reduced BP versus placebo; improvements in endothelial function and arterial stiffness were noted.	Mean 24 h SBP, mean 24 h DBP, office BP, endothelial function, arterial stiffness	Empagliflozin 25 mg/d	12 weeks	124	ChiCTR2100054678	(68)
Hypertension	Dapagliflozin significantly reduced albuminuria; effects remained after adjusting for changes in HbA1c, SBP, body weight, and eGFR.	Albuminuria, eGFR, HbA1c, SBP, body weight	Dapagliflozin 10 mg/d	12 weeks	356	NCT01137474, NCT01195662	(69)
Hypertension on background antihypertensive medication	Empagliflozin reduced SBP and DBP versus placebo, irrespective of the number of antihypertensives and use of diuretics or ACE inhibitors/ARBs.	Mean 24 h SBP, mean 24 h DBP, glycohemoglobin, body weight	Empagliflozin 10 or 25 mg/d	12 weeks	823	NCT01370005	(67)

To summarize, SGLT-2i such as empagliflozin and dapagliflozin provide significant benefits in managing hypertension in patients with T2DM. These benefits extend beyond BP control to include improvements in glycemic control, cardiovascular health, and renal protection, making them a valuable addition to the therapeutic arsenal for these patients.

### 3.4 Potential clinical applications and mechanisms of SGLT-2i for arrhythmias

Arrhythmia, also known as an irregular heartbeat, is a condition where the heart beats too fast, too slow, or with an irregular rhythm. Arrhythmias can originate from different parts of the heart, disrupting the normal organized and coordinated heartbeat (70). Frequent arrhythmias may indicate that the heart cannot pump enough blood to the body, which is crucial as the heart supplies nutrients and oxygen through the blood. This can lead to symptoms such as dizziness, fainting, or other signs of inadequate blood flow (71). Arrhythmias can be treated with medications or surgical procedures to control the irregular rhythm. AF is the most common type of treated heart arrhythmia. Without treatment, arrhythmias can damage the heart, brain, or other organs, potentially leading to life-threatening conditions like stroke, HF, or sudden cardiac arrest (72).

The anti-arrhythmic effects of SGLT-2i are mediated through various mechanisms. Primarily, these drugs reduce intracellular sodium and calcium overload, which helps stabilize cardiac myocytes and prevent arrhythmogenic events (73). SGLT-2i also promote glucosuria and natriuresis, leading to lower BP and reduced myocardial stress (74). Furthermore, these inhibitors improve myocardial energy metabolism, enhance mitochondrial function, and reduce oxidative stress and inflammation, all of which contribute to their anti-arrhythmic benefits (75). Additionally, SGLT-2i suppress sympathetic overactivity and RAAS activation, which further aids in preventing arrhythmias (76).

SGLT-2i have demonstrated significant potential in managing arrhythmias, particularly in patients with HF and T2DM. In patients with T2DM and arrhythmias, SGLT-2i have shown promising results. For example, in patients with T2DM and established CVD, empagliflozin significantly reduced cardiovascular death, HF hospitalizations, and renal events. The absolute reduction in these outcomes was greater in those with AF (77). For patients with HF, SGLT-2i have shown significant benefits in reducing arrhythmic events and improving outcomes. In HFrEF, dapagliflozin significantly reduced the risk of serious ventricular arrhythmias, cardiac arrest, or sudden death when added to conventional therapy (78). Dapagliflozin demonstrated consistent benefits across HFpEF and HFrEF, significantly reducing the risk of cardiovascular death or worsening HF, especially in patients with paroxysmal AF (79).

In addition, in patients treated with implantable cardioverter-defibrillator or cardiac resynchronization therapy-defibrillator, empagliflozin reduced the number of ventricular arrhythmias compared with placebo, indicating its efficacy in managing arrhythmic events in T2DM patients with implanted devices (80). Additionally, in T2DM patients with CKD, canagliflozin showed benefits in preventing hemorrhagic stroke and atrial fibrillation/flutter (AF/AFL), with significant protection observed in those with the lowest baseline eGFR (81). Table 4 summarizes clinical trials focused on SGLT-2i in arrhythmia.

**Table 4:** Overview of potential clinical trials investigating SGLT-2i in arrhythmia.

Disease characteristic	Primary finding	Measurement parameters	SGLT-2i dose	Follow-up duration	Patients enrolled	Clinical trial number	Refs.
Arrhythmias	Empagliflozin reduces the number of ventricular arrhythmias compared with placebo.	Number of ventricular arrhythmias, appropriate device discharges, blood ketones, hematocrit, brain natriuretic peptide, body weight	Empagliflozin once daily	24 weeks	150	jRCTs031180120	(80)
AF/AFL	Evidence of benefit in preventing AF/AFL	AF/AFL	Canagliflozin	Not specified	4,401	NCT02065791	(81)
HFpEF or HFrEF	Dapagliflozin showed consistent benefits across different types of AF, significantly reducing the risk of cardiovascular death or worsening HF, especially in patients with paroxysmal AF.	Composite of cardiovascular death or worsening HF, HF hospitalization rates, KCCQ scores.	Dapagliflozin	Not specified	6,263	NCT03619213	(79)
HFpEF	Empagliflozin effectively reduced cardiovascular death or HF hospitalization rates equally in patients with and without AF, also slowing eGFR decline in both groups.	Cardiovascular death, HF hospitalization rates, eGFR decline rate.	Empagliflozin	26 months	5,988	Not specified	(123)
HFrEF	Dapagliflozin reduced the risk of any serious ventricular arrhythmia, cardiac arrest, or sudden death when added to conventional therapy.	Incidence of serious ventricular arrhythmias, resuscitated cardiac arrest, sudden death	Dapagliflozin 10 mg/d	18.2 months (median)	4,744	NCT03036124	(78)
HFrEF	Dapagliflozin similarly reduced the risk of worsening HF or cardiovascular death in patients with and without AF, did not significantly impact new-onset AF.	Worsening HF events, cardiovascular death, all-cause mortality, KCCQ scores.	Dapagliflozin	18.2 months (median)	4,744	NCT03036124	(124)
Established CVD	Empagliflozin significantly reduced cardiovascular death, HF hospitalizations, and renal events in patients, with a greater absolute reduction in those with AF.	Cardiovascular death, HF hospitalization, new or worsening nephropathy, introduction of loop diuretics, occurrence of oedema.	Empagliflozin	Not specified	7,020	Not specified	(77)

In summary, SGLT-2i like ertugliflozin, dapagliflozin, and empagliflozin provide significant benefits in managing arrhythmias and improving cardiovascular outcomes in various patient populations, particularly those with HF and diabetes.

## 4 Clinical applications and mechanisms of SGLT-2i for CKD

CKD, also known as chronic renal failure, involves the gradual loss of kidney function. The kidneys filter waste and excess fluids from the blood, which are then excreted in the urine. In the early stages of CKD, there are usually few signs or symptoms. However, advanced CKD can lead to dangerous levels of fluid, electrolytes, and waste buildup in the body (82). Treatment for CKD focuses on slowing the progression of kidney damage, typically through managing the underlying cause (83). However, even with control of the underlying cause, kidney damage may continue to progress. CKD can eventually progress to end-stage renal disease, which can be life-threatening without dialysis or a kidney transplant (84).

**Figure 3:**
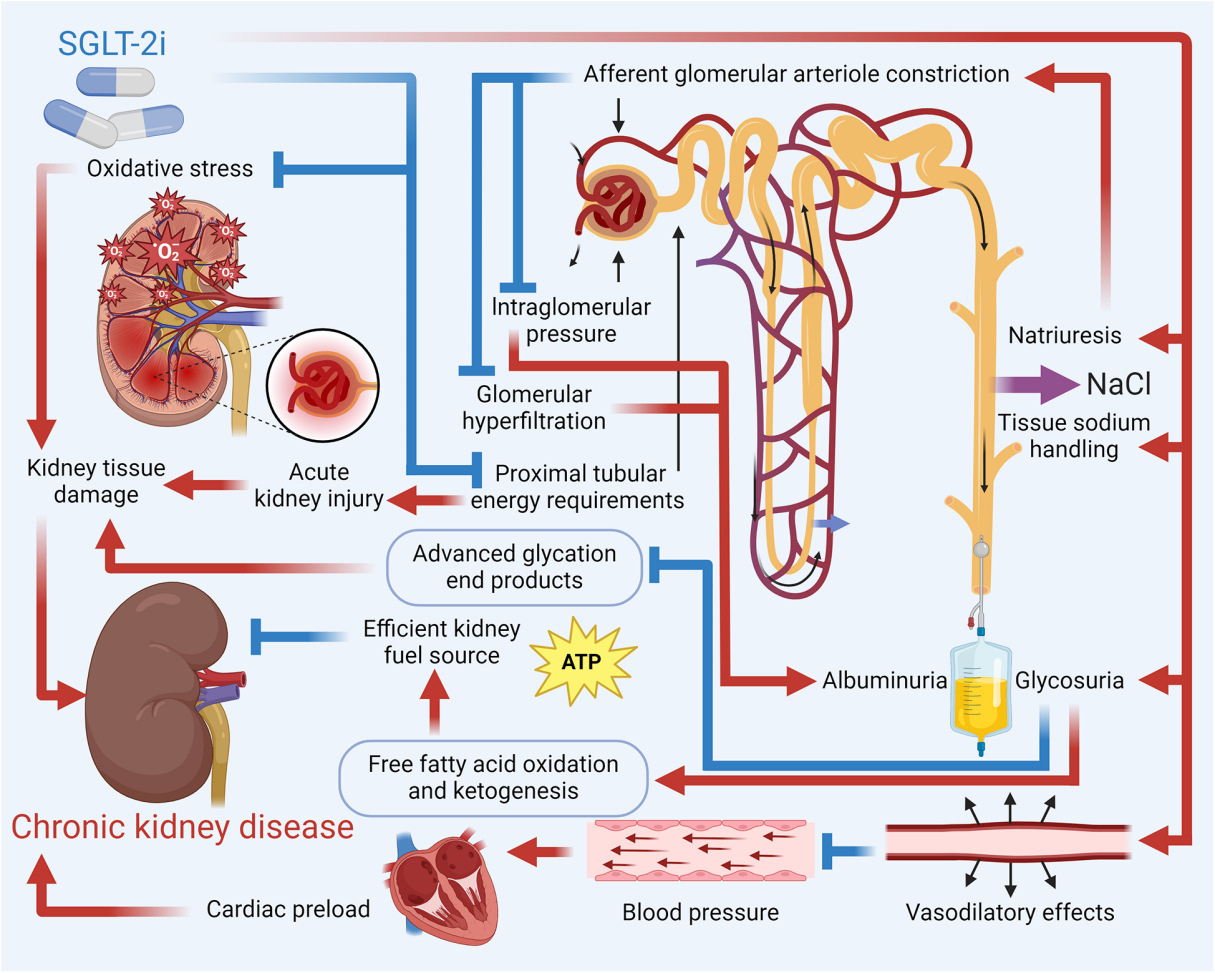
Mechanistic overview of SGLT-2i in reducing the risk of CKD. Key pathways by which SGLT-2i reduce the risk of CKD include enhanced natriuresis and improved tissue sodium handling that lower intraglomerular pressure and mitigate hyperfiltration; decreased cardiac preload and BP; and metabolic benefits such as increased ketogenesis and fatty acid oxidation. Additionally, these inhibitors reduce oxidative stress and inflammatory mediators, contributing to the prevention of kidney tissue damage and CKD progression.

SGLT2, located in the proximal convoluted tubule (S1 segment) of the nephron, is responsible for reabsorbing approximately 90% of the filtered glucose from the urine back into the bloodstream. It functions by coupling sodium (Na^+^) transport with glucose, utilizing the sodium gradient created by the Na^+^/K^+^ ATPase pump on the basolateral membrane, with glucose exiting the tubular cells into the bloodstream via GLUT2 transporters (85). SGLT-2i exert kidney-protective effects through multifaceted mechanisms that go beyond tubuloglomerular feedback-mediated reduction of intraglomerular pressure and hyperfiltration, a process pivotal in diabetic conditions through increased distal solute delivery to the macula densa, afferent arteriolar vasoconstriction, and efferent vasodilation. However, tubuloglomerular feedback's contribution varies with factors like salt intake, dietary protein, and underlying disease (e.g., weaker in non-diabetic CKD) (86). Additional mechanisms include optimization of energy metabolism, where glycosuria-induced caloric deficit promotes lipolysis, ketogenesis (β-hydroxybutyrate), and reduced proximal tubular oxygen consumption, mitigating hypoxia (87). SGLT-2i also enhance autophagy and cellular homeostasis by inhibiting mTORC1, activating AMPK/SIRT1 pathways, and balancing HIF-1α/HIF-2α signaling, reducing oxidative stress, inflammation, and fibrosis (88). They attenuate sympathetic hyperactivity via natriuresis, reduced Na^+^/H^+^ exchanger activity, and suppression of neurohormonal pathways, thereby lowering BP and cardiac strain (89). Furthermore, SGLT-2i improve vascular health by upregulating endothelial nitric oxide, reducing arterial stiffness, and mobilizing pro-vascular progenitor cells (90). These pleiotropic effects—alongside glycemic control, BP reduction, weight loss, and improved lipid profiles—contribute to a significant reduction in CKD progression, decrease albuminuria, and offer anti-inflammatory and antifibrotic benefits, highlighting SGLT-2 inhibitors' role beyond glucose-lowering. Figure 3 details how SGLT-2i help reduce the risk of CKD.

SGLT-2i have shown significant promise in managing CKD, particularly in patients with T2DM and those at high cardiovascular risk. Various clinical trials have demonstrated the renal and cardiovascular benefits of these inhibitors across different patient populations. Dapagliflozin therapy in patients with CKD, irrespective of T2DM status, led to a lower risk of kidney disease progression or death from cardiovascular causes (91). For patients with T2DM and chronic HF taking regular loop diuretics, empagliflozin caused a significant increase in 24 h urine volume without increasing urinary sodium. This combination therapy showed positive renal and cardiovascular effects, emphasizing the benefit of SGLT2 inhibition in conjunction with loop diuretics (39). Ertugliflozin was effective in reducing HbA1c, body weight, and SBP while maintaining eGFR in patients with T2DM and stage 3 CKD, proving to be well tolerated even in moderate-to-severe CKD (92). In patients without diabetes and with CKD, dapagliflozin exhibited a plasma concentration-time profile similar to that in diabetic kidney disease, with changes in risk markers for kidney disease, indicating its potential utility across different types of CKD (93).

Dapagliflozin has significantly reduced the risk of a composite of sustained decline in eGFRF, end-stage kidney disease, or death from renal or cardiovascular causes in patients with CKD (91). The DELIVER trial showed that dapagliflozin slowed the rate of decline in eGFR in patients with HFrEF or HFpEF (94). In a unique trial combining dapagliflozin with eplerenone, a robust additive HbA1c and urine albumin-creatinine ratio (UACR)-lowering effect was observed, suggesting potential synergistic benefits in CKD management (95). These findings highlight its role in mitigating disease progression and reducing severe renal and cardiovascular events. Canagliflozin has demonstrated a significant reduction in the risk of kidney failure and cardiovascular events in patients with T2DM and albuminuric CKD, making it an effective therapeutic option for protecting kidney function and cardiovascular health (96). Sotagliflozin, used in patients with diabetes and CKD, resulted in a lower risk of deaths from cardiovascular causes, hospitalizations for HF, and urgent HF visits, although it was associated with AEs (97). It also showed efficacy in reducing urine albumin-creatinine ratio (UACR) in patients with stage 3 and stage 4 CKD (98, 99). Table 5 highlights clinical trials assessing the impact of SGLT-2i in CKD.

**Table 5:** Overview of clinical trials investigating SGLT-2i in CDK.

Disease characteristic	Primary finding	Measurement parameters	SGLT-2i dose	Follow-up duration	Patients enrolled	Clinical trial number	Refs.
CKD without diabetes	Dapagliflozin plasma concentration-time profile in patients with non-diabetic kidney disease appears similar to the profile in diabetic kidney disease, with plasma exposure associated with changes in risk markers for kidney disease.	Plasma exposure, urinary albumin ratio, glomerular filtration rate, SBP	Dapagliflozin 10 mg/d	18 weeks	53	NCT03190694	(93)
CKD	Dapagliflozin significantly reduced the risk of a composite of a sustained decline in eGFR of at least 50%, end-stage kidney disease, or death from renal or cardiovascular causes compared with placebo.	eGFR, UACR, end-stage kidney disease, death from renal or cardiovascular causes, hospitalization for HF	Dapagliflozin 10 mg/d	2.4 years (median)	4,304	NCT03036150	(91)
CKD	Empagliflozin therapy led to a lower risk of progression of CKD or death from cardiovascular causes than placebo.	eGFR, UACR, end-stage kidney disease, death from renal causes, death from cardiovascular causes, hospitalization for HF	Empagliflozin 10 mg/d	2.0 years (median)	6,609	NCT03594110	(125)
CKD	Sotagliflozin resulted in a lower risk of the composite of deaths from cardiovascular causes, hospitalizations for HF, and urgent visits for HF than placebo but was associated with AEs.	Deaths from cardiovascular causes, hospitalizations for HF, urgent visits for heart failure, nonfatal MI, nonfatal stroke, AEs	Sotagliflozin 200 mg/d	16 months (median)	10,584	NCT03315143	(97)
CKD	BI 690517 dose-dependently reduced albuminuria with concurrent renin-angiotensin system inhibition and empagliflozin	UACR, hyperkalemia, adrenal insufficiency	Empagliflozin 10 mg/d	14 weeks	586	NCT05182840	(126)
CKD	Zibotentan combined with dapagliflozin reduced albuminuria with an acceptable tolerability and safety profile.	UACR, body weight, B-type natriuretic peptide, fluid retention	Dapagliflozin 10 mg/d	12 weeks	447	NCT04724837	(127)
Moderate-to-severe CKD	Dapagliflozin and dapagliflozin-saxagliptin reduced UACR versus placebo, with dapagliflozin-saxagliptin also reducing HbA1c. Both treatments were generally well tolerated.	UACR, HbA1c, body weight, SBP, AEs, serious AEs	Dapagliflozin 10 mg/d	24 weeks	461	NCT02547935	(128)
Stage 3 CKD	HbA1c was significantly reduced with sotagliflozin 400 mg compared with placebo in this CKD3 cohort. UACR in patients with albuminuria was reduced with each of the two doses.	HbA1c, fasting plasma glucose, body weight, SBP, UACR	Sotagliflozin 200 or 400 mg/d	52 weeks	787	NCT03242252	(98)
Stage 3 CKD	Ertugliflozin resulted in reductions in HbA1c, body weight, and SBP, maintenance of eGFR, and was generally well tolerated. Results in CKD stage 3B were similar except for an attenuated HbA1c response with the 15 mg dose.	HbA1c, body weight, SBP, eGFR, AEs, symptomatic hypoglycemia, hypovolemia, kidney-related AEs	Ertugliflozin 5 and 15 mg/d	18 weeks	1,776	NCT01986881	(92)
Stage 4 CKD	HbA1c reductions with sotagliflozin were not statistically significant versus placebo at 26 weeks, but were significant at 52 weeks. The 52-week safety profile was consistent with the SCORED outcomes trial.	HbA1c, body weight, SBP, eGFR, AEs	Sotagliflozin 200 or 400 mg/d	52 weeks	277	NCT03242018	(99)
Stage 4 CKD with albuminuria	The effects of dapagliflozin were consistent with those observed in the DAPA-CKD trial overall, with no evidence of increased risks.	GFR, urinary albumin-to-creatinine ratio, end-stage kidney disease, kidney or cardiovascular death, all-cause death	Dapagliflozin 10 mg/d	Not specified	624	NCT03036150	(129)
CKD with albuminuria	Canagliflozin significantly reduced the risk of kidney failure and cardiovascular events compared to placebo.	eGFR, serum creatinine level, UACR, end-stage kidney disease, death from renal or cardiovascular causes, hospitalization for HF, MI, stroke	Canagliflozin 100 mg/d	2.62 years (median)	4,401	NCT02065791	(96)
CKD with albuminuria	Combining dapagliflozin with eplerenone resulted in a robust additive UACR-lowering effect.	UACR, hyperkalemia, eGFR	Dapagliflozin 10 mg/d	8 weeks	46	EU 2017-004641-25	(95)
Diabetic kidney disease.	Dapagliflozin mitigated kidney function decline in patients with T2D at high cardiovascular risk.	eGFR, sustained decline ≥40% in eGFR to <60 ml/min/1.73 m^2^, end-stage kidney disease, kidney-related death, categorical eGFR reductions (≥57%, ≥50%, ≥40%, ≥30%)	Dapagliflozin 10 mg/d	Up to 4 years	15,201	NCT01730534	(130)
CKD with established CVD	Empagliflozin improved clinical outcomes and reduced mortality in vulnerable patients with T2DM, established CVD, and CKD.	Cardiovascular death, hospitalization for HF, all-cause hospitalization, all-cause mortality	Empagliflozin 10 or 25 mg/d	Not specified	7,020	NCT01131676	(131)
Chronic HF	Empagliflozin caused a significant increase in 24 h urine volume without an increase in urinary sodium when used in combination with loop diuretic.	24 h urinary volume, 24 h urinary sodium, fractional excretion of sodium, electrolyte-free water clearance, body weight, serum urate	Empagliflozin 25 mg/d	6 weeks	23	NCT03226457	(39)
HFrEF or HFpEF with an eGFR ≥25 ml/min/1.73 m^2^	Dapagliflozin did not significantly reduce the frequency of the kidney composite outcome, although the overall event rate was low. However, dapagliflozin slowed the rate of decline in eGFR compared with placebo.	Cardiovascular death, worsening HF, eGFR, first ≥50% decline in eGFR from baseline, first eGFR <15 ml/min/1.73 m^2^, end-stage kidney disease, death from kidney causes	Dapagliflozin 10 mg/d	2.3 years (median)	6,262	NCT03619213	(94)
HFrEF with an eGFR ≥30 ml/min/1.73 m^2^	The average dip in eGFR after dapagliflozin was started was small and associated with better clinical outcomes compared with a similar decline on placebo.	eGFR, primary outcome (worsening HF or cardiovascular death), >10%, >20%, >30% decline in eGFR, eGFR slopes	Dapagliflozin 10 mg/d	18.2 months (median)	4,744	NCT03036124	(132)

Collectively, SGLT-2i like empagliflozin, dapagliflozin, canagliflozin, and sotagliflozin offer significant benefits in managing CKD, from improving renal outcomes to reducing cardiovascular risks. Their application extends across various CKD patient profiles, highlighting their versatility and effectiveness in CKD management.

## 5 Potential clinical applications and mechanisms of SGLT-2i for NAFLD

NAFLD is a condition characterized by the accumulation of fat in the liver. It is most commonly found in individuals who are overweight or obese. With the rising prevalence of obesity, NAFLD is becoming increasingly common, especially in the Middle East and Western countries. It is now the most prevalent form of liver disease globally (100). The severity of NAFLD varies, ranging from steatosis to a more severe condition called non-alcoholic steatohepatitis (NASH). NASH causes liver inflammation and damage due to fat buildup in the liver. It can progress, leading to severe cirrhosis and even liver cancer. This damage is similar to that caused by heavy alcohol consumption (101).

**Figure 4:**
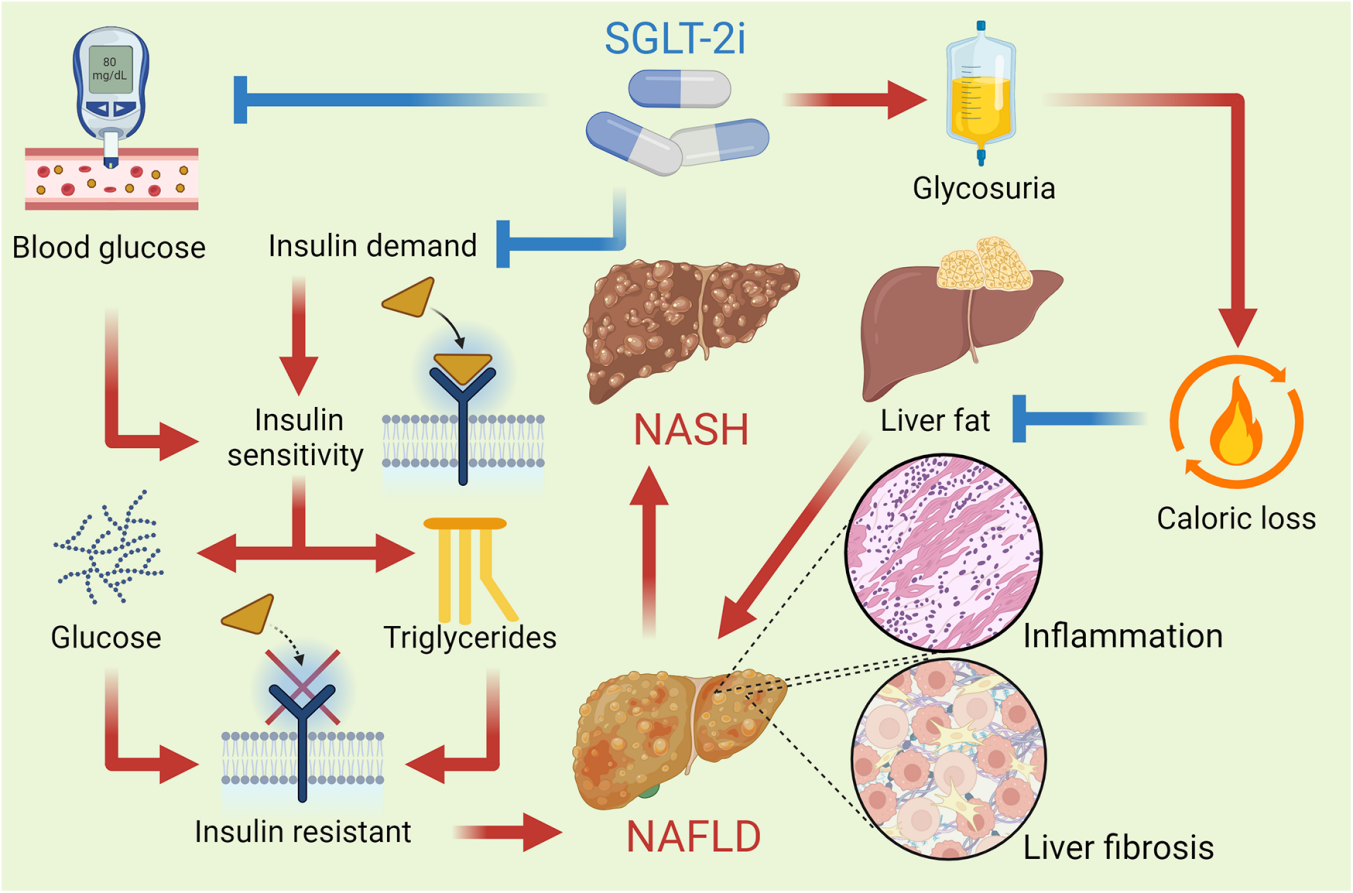
SGLT-2i improve insulin sensitivity by reducing blood glucose levels and promoting a lower insulin demand in the body. This improvement helps decrease hepatic glucose production and reduces the deposition of triglycerides in the liver. These drugs reduce the reabsorption of filtered glucose in the kidneys, leading to increased urinary glucose excretion and a consequent caloric loss, which promotes a reduction in the liver fat content. This reduction in hepatic steatosis is vital for improving liver function and reversing NAFLD. By reducing liver fat content, SGLT-2i also help lower the hepatic inflammation and fibrosis that characterize NASH, an advanced form of fatty liver disease.

Hepatic SGLT-2 expression is minimal, but SGLT-2i ameliorate NAFLD and NASH through indirect mechanisms, including reduced hepatic gluconeogenesis, improved insulin sensitivity, and modulation of adipokine signaling, such as increased adiponectin. These drugs primarily exert their effects in liver disease by improving glucose and lipid metabolism, reducing hepatic fat accumulation, and modulating inflammatory pathways (102). By inhibiting SGLT2 in the kidneys, SGLT-2i promote glucosuria, lowering blood glucose levels and insulin resistance, which can mitigate NAFLD. Additionally, SGLT-2i enhance ketogenesis, shifting the liver's energy metabolism from glucose to ketone bodies, potentially reducing hepatic steatosis. They also exert anti-inflammatory and antifibrotic effects by lowering pro-inflammatory cytokines and reducing oxidative stress, which further benefits liver function in chronic liver diseases. Furthermore, these inhibitors improve adiponectin levels, reduce inflammation, and enhance insulin sensitivity, all contributing to hepatoprotection (103). Overall, SGLT-2 inhibitors improve both metabolic and inflammatory parameters in the liver, offering potential therapeutic benefits for conditions such as NAFLD and NASH. Figure 4 describes the mechanisms by which SGLT-2i affect NAFLD.

SGLT-2i have shown significant potential in managing NAFLD. Various clinical trials have demonstrated the hepatic and metabolic benefits of these inhibitors across different patient populations. Empagliflozin and dapagliflozin have both shown efficacy in reducing liver fat and improving liver function. For instance, a trial involving dapagliflozin demonstrated improvements in liver dysfunction associated with a decrease in serum soluble dipeptidyl peptidase-4 (sDPP-4), suggesting a therapeutic strategy independent of glucose lowering or weight loss (104). Moreover, another study found dapagliflozin more beneficial than pioglitazone in patients with NAFLD, with improvements in fatty liver index closely related to glycemic control (105). Similarly, tofogliflozin was effective in reducing hepatic steatosis compared to pioglitazone, as indicated by lower MRI-proton density fat fraction (MRI-PDFF) levels (106). Additionally, lower serum high-molecular-weight (HMW) adiponectin was associated with a better response to dapagliflozin, indicating a potential biomarker for treatment efficacy (107). Furthermore, a study combining dapagliflozin and OM-3CA significantly reduced liver fat content, with dapagliflozin monotherapy reducing hepatocyte injury biomarkers and FGF21 (108). Canagliflozin also reduced body mass, fat mass, and hepatic fat content without significantly reducing muscle mass, demonstrating its effectiveness in altering body composition favorably (109).

In patients with T2DM and NAFLD, empagliflozin significantly improved hepatic steatosis compared with sitagliptin, potentially protecting against hepatic insulin resistance (110). Similarly, dapagliflozin improved liver steatosis and attenuated liver fibrosis in patients with significant liver fibrosis, highlighting its potential for treating liver conditions associated with NAFLD (111). Dapagliflozin also decreased liver fat content and pancreatic fat content while improving serum ALT, TNF-α, and IL-6 levels, indicating broad metabolic benefits (112). Furthermore, in non-insulin-dependent T2DM patients with NAFLD, dapagliflozin significantly decreased intrahepatic lipid content, body weight, body fat, and visceral fat/subcutaneous fat ratio, along with improvements in HbA1c and ALT levels (113). Additionally, in patients with NAFLD without diabetes, empagliflozin improved liver steatosis and measures of liver fibrosis, suggesting its applicability in non-diabetic populations as well (114). Table 6 discusses clinical trials exploring the effects of SGLT-2i on NAFLD.

**Table 6:** Overview of potential clinical trials investigating SGLT-2i in NAFLD.

Disease characteristic	Primary finding	Measurement parameters	SGLT-2i dose	Follow-up duration	Patients enrolled	Clinical trial number	Refs.
NAFLD without T2DM	Empagliflozin improves liver steatosis and, more importantly, measures of liver fibrosis in patients with NAFLD without T2DM.	CAP score, liver stiffness measurement, AST, ALT, fasting insulin levels, visual analysis of ultrasound images	Empagliflozin 10 mg once daily	24 weeks	90	IRCT20190122042450N1	(114)
NAFLD	Empagliflozin significantly improves hepatic steatosis compared with sitagliptin, potentially protecting against hepatic insulin resistance.	Intrahepatic lipid content, intramuscular and extramuscular lipid content, body composition, tissue-specific insulin sensitivity	Empagliflozin 10 mg/day	12 weeks	44	Not specified	(110)
NAFLD	Dapagliflozin improves liver steatosis and attenuates liver fibrosis in patients with significant liver fibrosis.	Controlled attenuation parameter (CAP), liver stiffness measurement, serum alanine aminotransferase (ALT), γ-glutamyltranspeptidase levels, visceral fat mass	Dapagliflozin 5 mg/day	24 weeks	57	Not specified	(111)
NAFLD	Dapagliflozin significantly decreases liver fat content and pancreatic fat content and improves serum ALT, TNF-α, and IL-6 levels.	Liver fat content, pancreatic fat content, liver fibrosis index, inflammatory cytokine levels, liver enzyme levels	Dapagliflozin	24 weeks	84	ChiCTR2100054612	(112)
NAFLD	Dapagliflozin improves liver dysfunction associated with a decrease in serum sDPP-4, suggesting a therapeutic strategy independent of glucose lowering or weight loss.	Serum levels of sDPP-4, Visceral adipose tissues and subcutaneous adipose tissues, liver enzymes	Dapagliflozin 5 mg/day	24 weeks	57	Not specified	(104)
NAFLD	Dapagliflozin is more beneficial than pioglitazone in patients with NAFLD.	Fatty liver index, glycated hemoglobin, insulin level	Dapagliflozin	24 weeks	53	UMIN000022804	(105)
NAFLD	Tofogliflozin reduced MRI-PDFF levels, indicating a decrease in hepatic steatosis, compared to pioglitazone.	MRI-PDFF, body weight	Tofogliflozin 20 mg	24 weeks	40	jRCTs031180159	(106)
NAFLD	Lower serum HMW adiponectin is associated with a better response to dapagliflozin.	Serum HMW adiponectin, visceral fat area, hepatic steatosis, HbA1c, CAP	Dapagliflozin 5 mg/day	24 weeks	57	UMIN000022155	(107)
NAFLD	Dapagliflozin significantly decreases intrahepatic lipid contents, bodyweight, body fat, visceral fat/subcutaneous fat ratio, HbA1c, and ALT.	Intrahepatic lipid contents, liver attenuation index, bodyweight, body fat, visceral fat/subcutaneous fat ratio, HbA1c, ALT, adipokines	Dapagliflozin 10 mg/day	12 weeks	38	TCTR20170511001	(113)
NAFLD	Combined treatment with dapagliflozin and OM-3CA significantly reduced liver fat content. Dapagliflozin monotherapy reduced all measured hepatocyte injury biomarkers and FGF21.	MRI-PDFF, liver volume, glucose and lipid metabolism markers, hepatocyte injury and oxidative stress markers	Dapagliflozin 10 mg/day	12 weeks	84	NCT02279407	(108)
NAFLD	Canagliflozin reduced body mass, fat mass and hepatic fat content without significantly reducing muscle mass.	Body composition measured by bioelectrical impedance analysis method, hepatic fat fraction measured by MRI, serological markers	Canagliflozin 100 mg/day	12 months	124	UMIN000020615	(109)
NAFLD	Dapagliflozin and pioglitazone exerted beneficial effects on NAFLD through different mechanisms.	Liver-to-spleen ratio, bodyweight, visceral fat area, serum adiponectin levels, hyperglycemia	Dapagliflozin 10 mg/d	28 weeks	98	UMIN 000021291	(133)

In conclusion, SGLT-2i like empagliflozin, dapagliflozin, tofogliflozin, and canagliflozin provide significant benefits in managing NAFLD by improving liver steatosis, reducing liver fibrosis, and enhancing metabolic health, particularly in patients with T2DM. These findings highlight their potential as effective therapeutic options for NAFLD management.

## 6 Limitation and future perspective

Most trials show consistent benefits. For example, the DAPA-MI trial (NCT04564742) investigates dapagliflozin in post-MI patients without diabetes, while EMBODY (NCT04193557) explores empagliflozin's effects on cardiac nerve activity. Upcoming trials like ZENITH-CKD (NCT04724837) evaluate combination therapies (SGLT-2i + endothelin antagonists) for advanced CKD. However, some studies report neutral effects on arrhythmias or variable efficacy in non-diabetic NAFLD. For instance, dapagliflozin improved liver steatosis in diabetic patients but showed modest effects in non-diabetic cohorts (115). Despite robust cardiorenal benefits, SGLT-2i are not universally effective. Genitourinary infections, volume depletion risks, and incomplete responses in high-risk subgroups (e.g., advanced CKD) necessitate personalized approaches. Therefore, further research is needed to overcome the therapeutic limitations of SGLT-2i.

Firstly, while the cardiovascular and renal benefits of these inhibitors are well-documented, their long-term safety and efficacy across diverse patient populations require continued scrutiny. Clinical trials have predominantly focused on patients with T2DM, CVD, CKD, and NAFLD necessitating studies in broader cohorts to validate their utility in other conditions.

Additionally, the mechanisms underpinning the multifaceted effects of SGLT-2i are complex and not fully understood. While current evidence suggests roles in modulating glucose metabolism, reducing inflammation, and improving cardiovascular and renal function, further research is needed to elucidate these pathways and identify potential biomarkers for patient stratification and treatment optimization.

Another limitation is the potential for adverse effects, including genitourinary infections and dehydration, particularly in elderly patients or those with compromised renal function (116). To mitigate these risks, careful patient selection and individualized treatment strategies are essential. Baseline renal function, fluid status, and infection history should be evaluated before initiating therapy. Patient education on maintaining proper hydration, recognizing early signs of dehydration or infection, and practicing good hygiene can further minimize complications. Close monitoring of renal function, electrolyte balance, and hemodynamic status, particularly in those on concomitant diuretics or RAAS inhibitors, is recommended to prevent excessive volume depletion.

While SGLT-2i provide significant cardiovascular and renal protection, these benefits may not be sufficient for all patients, especially those with high cardiovascular risk profiles. In such patients, the protective effects of SGLT-2i alone might not fully address the complex cardiovascular challenges they face. Therefore, combining SGLT-2i with other guideline-directed medical therapies (GDMT) is recommended. GDMT typically includes medications such as ACE inhibitors, angiotensin II receptor blockers, beta-blockers, and mineralocorticoid receptor antagonists, which have been proven to improve HF outcomes and reduce cardiovascular events. This combination approach, by targeting multiple disease pathways, can offer more robust protection against heart attacks, strokes, and other complications, thereby optimizing cardiovascular outcomes. Thus, while SGLT-2i are an essential part of managing HF and CKD, their limitations highlight the need for their use within the broader context of GDMT.

**Figure 5:**
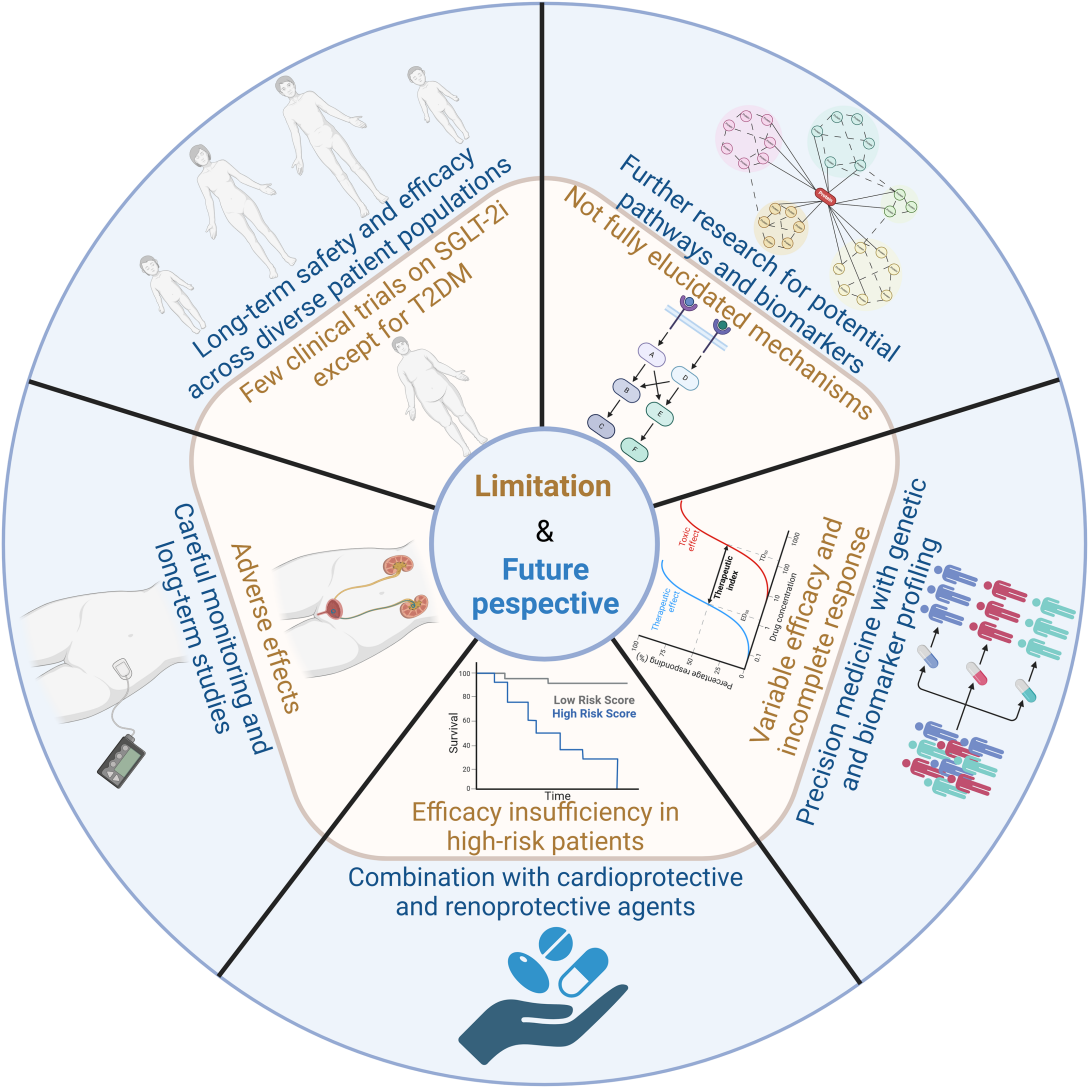
Limitations and future perspectives of SGLT-2i. The key challenges and future directions in the clinical application of SGLT2 inhibitors includes long-term safety and efficacy across diverse patient populations, further research for potential pathways and biomarkers, variable efficacy and incomplete responses, combination with cardioprotective and renoprotective agents, adverse effects and careful monitoring.

SGLT-2i do not elicit a complete response in all patients, and the therapeutic efficacy can vary significantly between individuals. This variability underscores the need for more personalized approaches to treatment. The integration of precision medicine strategies, such as genetic and biomarker profiling, holds promise for tailoring SGLT-2i therapy to the specific needs of individual patients. By identifying genetic factors and biomarkers that predict response, healthcare providers can better optimize treatment plans, potentially improving therapeutic outcomes and minimizing the risks of adverse effects. This personalized approach could enhance the overall effectiveness of SGLT-2i across diverse patient populations. Figure 5 outlines the limitations and future perspectives of SGLT-2i.

In the future, the clinical significance of SGLT-2 inhibitors is expected to expand, particularly in the management of cardiovascular, renal, and metabolic diseases such as NAFLD. As ongoing trials explore their potential in non-diabetic populations, SGLT-2 inhibitors may become a key treatment option for multi-system diseases. Additionally, the development of personalized medicine, through genetic profiling and biomarker identification, will likely enhance their efficacy across diverse patient groups. Long-term studies will further evaluate their enduring benefits in reducing cardiovascular and renal events, solidifying their role in improving patient outcomes and quality of life.

## 7 Conclusions

SGLT-2i have revolutionized the management of not only T2DM but also HF and CKD, among other conditions. This review underscores their broad therapeutic potential, highlighting their beneficial effects in MI, hypertension, arrhythmias, and NAFLD. The multifaceted mechanisms of SGLT-2i, including glucose excretion, reduction of oxidative stress and inflammation, improved myocardial and renal function, and modulation of neurohormonal pathways, contribute to their efficacy. As we continue to explore the full spectrum of SGLT-2i applications, these inhibitors hold promise for significantly improving patient outcomes across a range of cardiovascular, renal, and metabolic diseases.
